# Prognostic effect of high-flux hemodialysis in patients with chronic kidney disease

**DOI:** 10.1590/1414-431X20154708

**Published:** 2015-11-27

**Authors:** X. Li, H. Xu, X.C. Xiao, S.L. Deng, W. Wang, R. Tang

**Affiliations:** 1 The Hemodialysis Room, Xiangya Hospital of Central South University, Changsha, P.R., China; 2 Department of Educational Administration, Xiangya Hospital of Central South University, Changsha, P.R., China

**Keywords:** High-flux hemodialysis, Low-flux hemodialysis, Chronic kidney disease, Chronic renal failure, All-cause death rate, Cardiovascular death rate

## Abstract

We investigated the prognostic effects of high-flux hemodialysis (HFHD) and low-flux hemodialysis (LFHD) in patients with chronic kidney disease (CKD). Both an electronic and a manual search were performed based on our rigorous inclusion and exclusion criteria to retrieve high-quality, relevant clinical studies from various scientific literature databases. Comprehensive meta-analysis 2.0 (CMA 2.0) was used for the quantitative analysis. We initially retrieved 227 studies from the database search. Following a multi-step screening process, eight high-quality studies were selected for our meta-analysis. These eight studies included 4967 patients with CKD (2416 patients in the HFHD group, 2551 patients in the LFHD group). The results of our meta-analysis showed that the all-cause death rate in the HFHD group was significantly lower than that in the LFHD group (OR=0.704, 95%CI=0.533-0.929, P*=*0.013). Additionally, the cardiovascular death rate in the HFHD group was significantly lower than that in the LFHD group (OR=0.731, 95%CI=0.616-0.866, P<0.001). The results of this meta-analysis clearly showed that HFHD decreases all-cause death and cardiovascular death rates in patients with CKD and that HFHD can therefore be implemented as one of the first therapy choices for CKD.

## Introduction

Chronic kidney disease (CKD), which may lead to chronic renal failure, is characterized by a gradual loss in renal function over a period of months to years. This loss in renal function is defined by a persistent reduction in the glomerular filtration rate (GFR) or functional or structural abnormalities of kidneys on biopsy, urinalysis, or imaging (1). The GFR plays a crucial role in CKD and is used to classify the disease into five stages according to the Kidney Disease: Improving Global Outcomes (KDIGO) guidelines: >90 mL/min per 1.73 m^2^ (stage 1), 60-89 mL/min per 1.73 m^2^(stage 2), 30-59 mL/min per 1.73 m^2^ (stage 3), 15-29 mL/min per 1.73 m^2^ (stage 4), and <15 mL/min per 1.73 m^2^ (stage 5, or end-stage renal disease) (2,3). Increasing morbidity, high expense, and poor outcomes of CKD have caused it to become a well-known health threat (4). The prevalence of CKD is >200 cases per 1 million people per year, and nearly 400 cases are diagnosed each year in the US and Taiwan (2). The risk factors for CKD include age, hypotension, cardiac dysfunction, diabetes mellitus, obesity, atherosclerosis, and nephrotoxic drugs (5,6). Risk factors for end-stage renal disease include hypotension, age, daily proteinuria, a history of chronic renal insufficiency, a family history of kidney disease, obesity, hyperuricemia, diabetes mellitus, and heroin abuse (7). With respect to treatment of CKD, stages 1 to 4 require broad management principles such as blood pressure control and treatment of the primary disease, and stage 5 CKD usually requires renal replacement treatment (including dialysis), and transplantation is recommended when the renal function is insufficient to maintain health (8,9).

Hemodialysis (HD) utilizes countercurrent flow to achieve extracorporeal removal of waste products from blood, including urea, creatinine, and free water, when the kidneys are in a state of failure (10). Dialyzers are a part of the filter equipment used in HD; their hollow fiber walls are made of a semipermeable membrane. High-flux HD (HFHD) and low-flux HD (LFHD) are distinguished based on the pore size and fiber area (http://www.google.com.proxy.its.virginia.edu/patents/US20110009), which allows effective removal of uremic toxins and fluids. HD procedures are routinely patient-specific and involve a detailed prescription by a nephrologist, including frequency, length of each treatment, flow rates of the blood and dialysis solution, and dialyzer size (11). Complications associated with HD include chest pain, low blood pressure, fatigue, nausea, leg cramps, and headache (12). Contrasting opinions exist among experts regarding the procedure, with multiple studies confirming the benefits of HFHD in treating CKD (8,13) and other studies questioning its benefits (14,15). To resolve this issue, we conducted the present meta-analysis to investigate the prognostic effect of HFHD in patients with CKD.

## Material and Methods

### Data sources and key words

Computerized bibliographic databases were searched to identify relevant studies on the prognostic effect of HFHD in patients with CKD, without restrictions on data collection. The following databases were searched: PubMed, Springerlink, Wiley, EBSCO, Ovid, Web of Science, Wanfang database, China National Knowledge Infrastructure (CNKI), and the Weipu Journal Database. The following combination of free words and key words was applied in our rigorous search strategy: “high flux hemodialysis” or “high-flux hemodialysis” or “high permeable hemodialysis” or “HFHD.” Furthermore, manual searches were applied to identify additional potentially relevant articles.

### Inclusion and exclusion criteria

Published studies eligible for enrollment in the current meta-analysis were required to fulfill the following selection criteria: 1) research type: case-control studies comparing the prognostic effects of HFHD and LFHD in patients with CKD; 2) research objective: all patients with CKD were treated by HD; and 3) end outcomes: the all-cause death rate among patients with CKD and the cardiovascular death rate. Studies were excluded if 1) they had incomplete data; 2) they were published repeatedly; or 3) the diagnostic criteria were unclear.

### Data extraction and quality assessment

Two reviewers independently extracted data from all enrolled studies using a standardized data-extraction form and reached an agreement on all items after discussion. The following information was collected: surname of first author, time of publication, country, ethnicity, language, disease, age, gender, sample size, follow-up time and study design. The quality of enrolled studies was independently evaluated by two reviewers based on the Critical Appraisal Skills Programme (CASP) criteria (http://www.casp-uk.net/). The CASP criteria are scored based on the following 12 aspects: Does the study address a clearly focused issue (CASP01)? Was the recruited cohort selected in an acceptable way (CASP02)? Was the exposure accurately measured to minimize bias (CASP03)? Was the outcome accurately measured to minimize bias (CASP04)? Have the authors identified all important confounding factors (CASP05)? Was the follow-up of subjects complete enough (CASP06)? What are the results of the study (CASP07)? How precise are the results (CASP08)? Are the results believable (CASP09)? Can the results be applied to the local population (CASP10)? Do the results of the study fit with other available evidence (CASP11)? What are the implications of this study for practice (CASP12)?

### Statistical analysis

Comprehensive meta-analysis 2.0 (CMA 2.0; https://www.meta-analysis.com/downloads/Meta-Analysis-Manual.pdf) was applied in our meta-analysis. The prognostic effects of HFHD and LFHD in patients with CKD were evaluated by odds ratios (ORs) and their 95% confidence intervals (95%CIs) under a fixed-effects or random-effects model. The significance of pooled standardized mean differences was detected by the Z-test. Cochran’s Q-statistic (P*<*0.05 was considered significant) and the *I* ^2^ test (0%, no heterogeneity; 100%, maximal heterogeneity) were also applied to reflect the heterogeneity among studies (16). A random-effects model was used if there was evidence of significant heterogeneity (P*<*0.05 or *I* ^2^ test exhibited >50%); otherwise, a fixed-effects model was applied (17). The potential source of heterogeneity was assessed by univariate and multivariate meta-regression analysis, and Monte Carlo simulation was conducted for reexamination (16,18,19). Sensitivity analysis was conducted by deleting each enrolled study to estimate the effect of a single study on the overall results. A funnel plot, the classic fail-safe N method, and the Egger test were implemented to assess whether publication bias existed and thus further confirm the original result (20,21). All tests were two-sided, and a P value of *<*0.05 was considered to be statistically significant.

## Results

### Baseline characteristics

Based on our rigorous criteria, we retrieved 227 studies through both electronic database and manual searching. The retrieved studies were carefully screened to exclude duplicates (n=20), letters, reviews, and meta-analyses (n=6); non-human studies (n=25); and studies not related to HD (n=42). The full texts of the remaining studies (n=134) were reviewed, and additional studies were excluded if they were not relevant to HFHD (n=37) or LFHD (n=41) or lacked data related to mortality (n=44). After the remaining 12 trials were further assessed, eight eligible cohort studies performed from 2002 to 2013 were finally selected for the present meta-analysis. The selected studies involved 4967 patients with CKD (2416 in the HFHD group, 2551 in the LFHD group) (13 15 22 23 24 25 26). All eight studies involved Caucasians; two were from the US, one was from the UK, one was from Belgium, one was from Sweden, two were from Germany, and one was from France. The sample size of all enrolled studies ranged from 64 to 1846. The baseline characteristics of the eight studies are shown in Table 1.

**Table:**
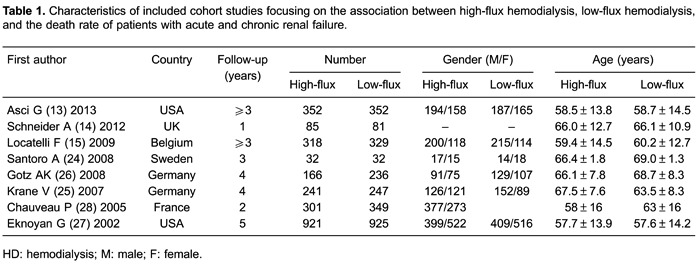

### Pooled outcome of meta-analysis

**Figure 1:**
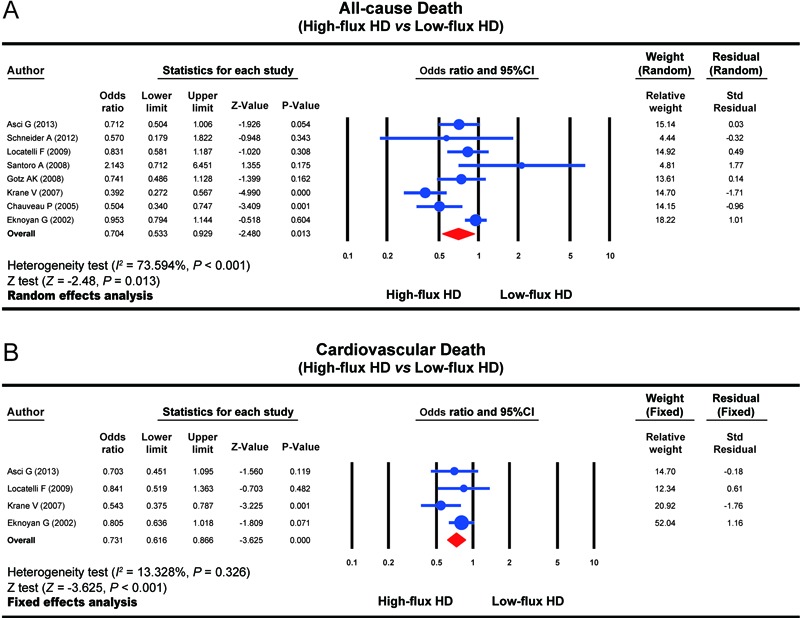
Forest plots of the influence of high-flux hemodialysis and low-flux hemodialysis on the all-cause death rate and cardiovascular death rate of patients with chronic renal disease. HD: hemodialysis. See Table 1 for reference numbers.

The influence of HFHD and LFHD on the all-cause death rate among patients with CKD was reported by all eight enrolled studies. A random-effects model was applied because of the presence of heterogeneity among studies (*I* ^2^=73.594%, P*<*0.001). The results of our meta-analysis showed that the all-cause death rate in the HFHD group was evidently lower than that in the LFHD group (OR=0.704, 95%CI=0.533-0.929, P*=*0.013) (Figure 1A).

Four studies reported the influence of HFHD and LFHD on the cardiovascular death rate among patients with CKD. A fixed-effects model was applied because of the absence of heterogeneity among studies (*I* ^2^=13.328%, P=0.326). The results of our meta-analysis showed that the cardiovascular death rate in the HFHD group was significantly lower than that in the LFHD group (OR=0.731, 95%CI=0.616-0.866, P*<*0.001) (Figure 1B).

**Figure 2:**
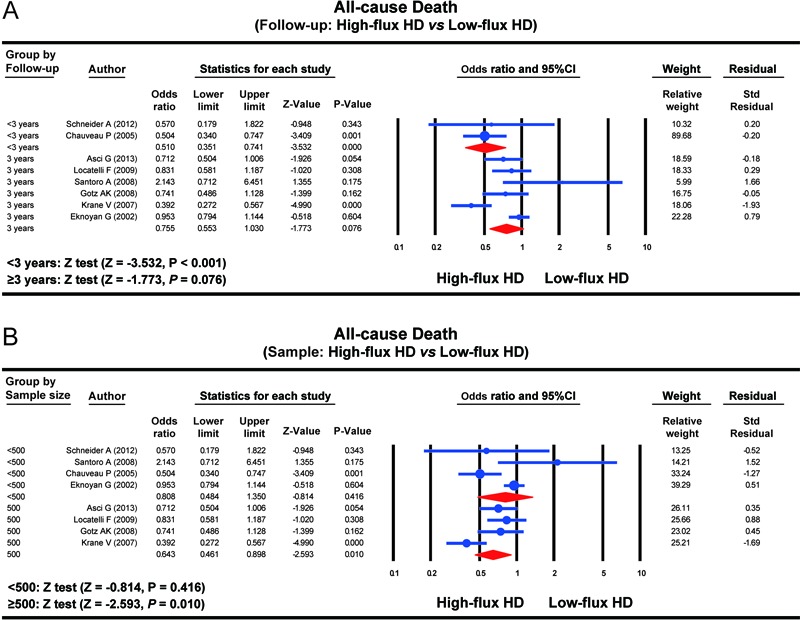
Forest plots of the influence of high-flux hemodialysis and low-flux hemodialysis on the all-cause death rate of patients with chronic renal disease in subgroup analyses. HD: hemodialysis. See Table 1 for reference numbers.

Subgroup analysis based on the follow-up demonstrated that the all-cause death rate in the HFHD group was evidently lower than that in the LFHD group within a follow-up of <3 years (OR=0.510, 95%CI=0.351-0.741, P*<*0.001); however, the difference in the all-cause death rate between the HFHD and LFHD group exhibited no statistical significance with a follow-up of ≥3 years (OR=0.755, 95%CI=0.553-1.030, P*=*0.076) (Figure 2A). A subgroup analysis based on sample size clarified that the all-cause death rate in the HFHD group was evidently lower than that in the LFHD group (n≥500) (OR=0.643, 95%CI=0.461-0.898, P*=*0.010), but the difference in the all-cause death rate between the HFHD and LFHD groups showed no statistical significance (n*<*500) (OR=0.808, 95%CI=0.484-1.350, P*=*0.416) (Figure 2B).

### Sensitivity analysis and publication bias

**Figure 3:**
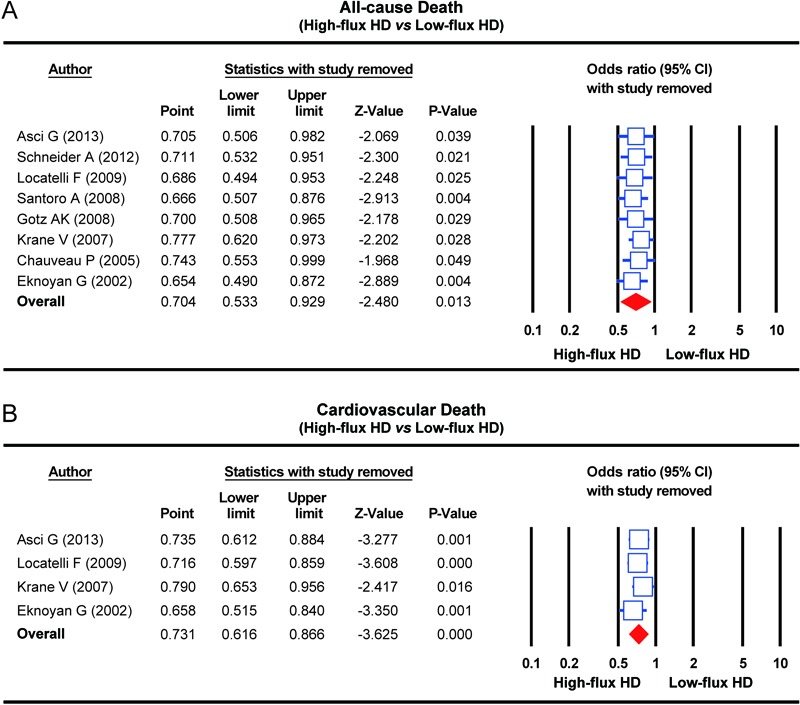
Sensitivity analyses of the influence of high-flux hemodialysis and low-flux hemodialysis on the all-cause death rate and cardiovascular death rate of patients with chronic renal disease. HD: hemodialysis. See Table 1 for reference numbers.

**Figure 4:**
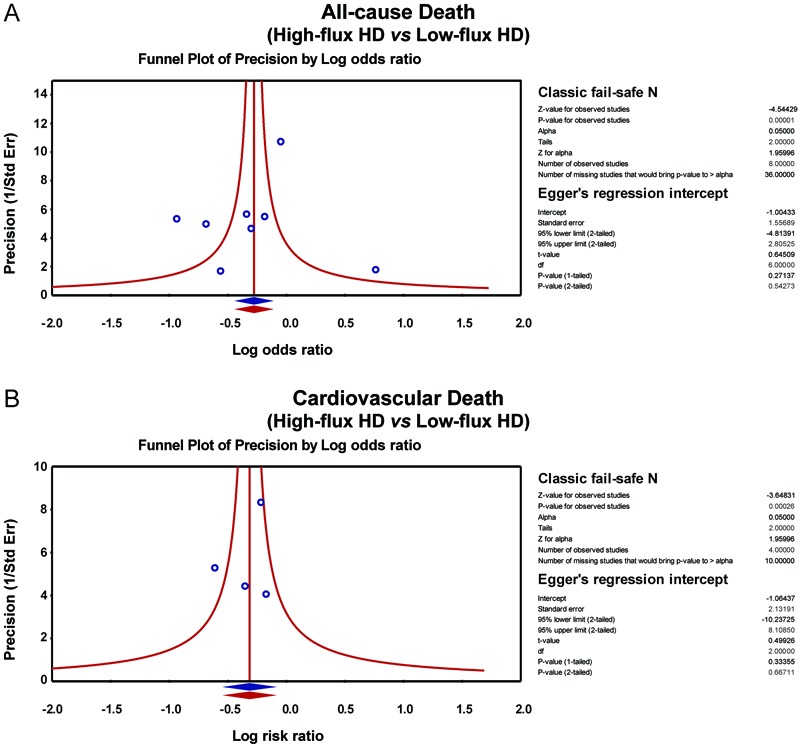
Publication biases of the influence of high-flux hemodialysis and low-flux hemodialysis on the all-cause death rate and cardiovascular death rate of patients with chronic renal disease. HD: hemodialysis. See Table 1 for reference numbers.

**Figure 5:**
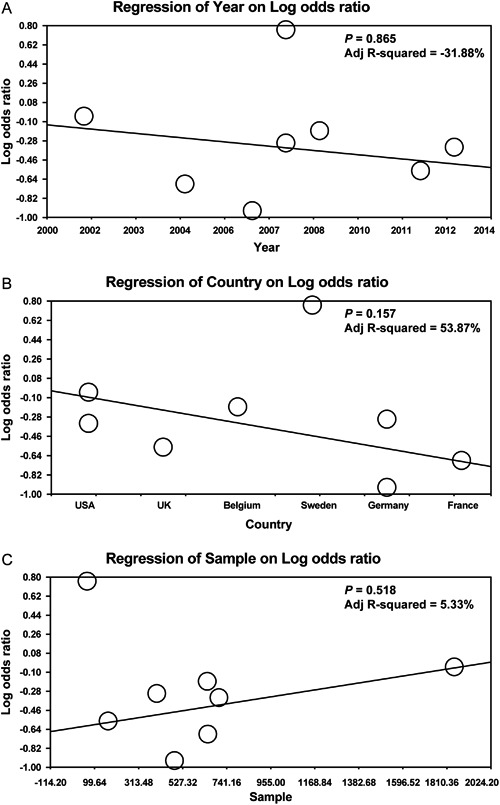
Meta-regression analyses of the influence of high-flux hemodialysis and low-flux hemodialysis on the all-cause death rate of patients with chronic renal disease by year, country, and sample. See Table 1 for reference numbers.

The result of the sensitivity analysis showed that none of the enrolled studies had a significant effect on the pooled standardized mean differences for the influence of HFHD and LFHD to the all-cause death rate and cardiovascular death rate in patients with CKD (Figure 3). The symmetrical funnel plots suggested that there was no publication bias in the enrolled studies, and the Egger linear regression analysis and classic fail-safe N method further confirmed the lack of publication bias (all P>0.05) (Figure 4). Univariate and multivariate meta-regression analysis showed that the publication year, country, and sample size were not potential sources of heterogeneity or crucial factors influencing the overall effect size (all P>0.05) (Figure 5, Table 2).

**Table:**
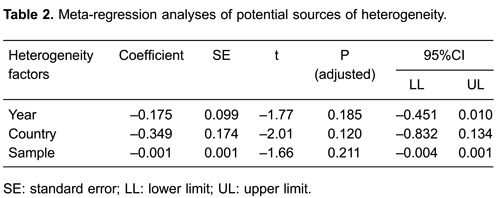

## Discussion

We performed a systematic meta-analysis to investigate the prognostic effect of HFHD and LFHD in patients with CKD. The main results of our meta-analysis revealed that the all-cause death rate and cardiovascular death rate in the HFHD group were remarkably lower than those in the LFHD group, suggesting that HFHD can be implemented as the first-line therapy choice in patients with CKD. In renal failure, the kidneys fail to filter the blood from water. Such failure can be caused by many factors including drug overdoses, crush syndrome, uncontrolled hypertension, long-term diabetes, and genetic predisposition such as that caused by *APOL1* mutation (27,28). CKD, a growing public health problem, is commonly characterized by albuminuria and/or a reduced GFR, which plays a crucial role in evaluating renal function (29). HD is the most common treatment for CKD and efficiently cleans the blood outside the body in an artificial kidney using a dialysis machine (30). HFHD is an extracorporeal blood cleansing process that is mainly useful in eliminating or clearing small-molecular-weight solutes similar to creatinine and urea, for which diffusive mass transfer is swift (31). HFHD is performed using a high-flux biocompatible dialyzer and can minimize inflammation and oxidative stress and improve the survival rate and quality of life of patients with CKD (32 33 34). HFHD involves the use of dialyzer membranes with notable porosity to larger molecules ([beta-2 microglobulin (β2-M)] clearance of >20 mL/min) following an increase in the ultrafiltration coefficient to >15 mL/mmHg per hour, which has better biocompatibility and an amelioration in middle-to-large molecule clearance with subsequent reduction in the residual uremic milieu (35). β2-M, the non-polymorphic chain, is found on the surface of all nucleated cells with a normal synthesis rate of 2 to 4 mg/kg per day, which varies inversely with the GFR (36). Filtered by the glomerulus, β2-M is also decreased by HFHD treatment, and this is beneficial to patients in delaying amyloid-related arthropathy (37,38). In accordance with our main results, Cheung et al. also found that the serum β2-M level was significantly lower with utilization of HF dialyzers than with LF dialyzers because of the presence of 12,000-Da molecules, which LF dialyzers cannot clear (39). Patients with lipid metabolism disorders undergoing HD also exhibited improvement in their symptoms following HFHD treatment. Such treatment is also associated with decreased complications of cardiovascular diseases (40).

The subgroup analysis based on follow-up revealed that the all-cause death rate in the HFHD group was significantly lower than that in the LFHD group within a follow-up of <3 years, and the difference in the all-cause death rate between the HFHD and LFHD groups was not statistically significant within a follow-up of ≥3 years. A subgroup analysis based on sample size showed that the all-cause death rate in the HFHD group was lower than that in the LFHD group, but the difference in the all-cause death rate between the HFHD and LFHD groups was not statistically significant.

Potential limitations of this study should be taken into consideration. First and foremost, only Caucasian patients were analyzed; this might have contributed to selection bias. Another important limitation was that language bias might have been present because all studies were published only in the English language. Additionally, the absence of data on end outcomes may have resulted in questionable validity of our results. Finally, the enrolled studies did not provide detailed data on clinical subtypes; thus, further investigation of subtypes by subgroup analysis could not be performed.

In summary, our meta-analysis provides strong evidence that HFHD can decrease the all-cause death rate and cardiovascular death rate in patients with CKD, and HFHD can be implemented as a first-line therapy choice for CKD. However, future studies with larger populations, diverse ethnicities, and better study designs are required for a comprehensive analysis of the benefits of HFHD in patients with CKD.
